# O03 Review of a formalized palliative care OPAT service in a London teaching hospital

**DOI:** 10.1093/jacamr/dlaf239.003

**Published:** 2026-01-05

**Authors:** Elena Ferran, Hannah Kershaw, Antonia Scobie, Philip Lodge, Sophie Collier

**Affiliations:** The Royal Free NHS Foundation Trust, London, UK; The Royal Free NHS Foundation Trust, London, UK; The Royal Free NHS Foundation Trust, London, UK; The Royal Free NHS Foundation Trust, London, UK; The Royal Free NHS Foundation Trust, London, UK; University College London, London, UK

## Abstract

**Background:**

Outpatient Parenteral Antimicrobial Therapy (OPAT) is an established model of care in the UK. The 2019 BSAC guidance recognized the role of *palliative OPAT*, where the focus shifts from cure to quality of life. Published evidence remains limited, with Hart *et al.* (2020) reporting nine patients showing a clinical need. The Royal Free Hospital, a tertiary teaching hospital in London, established a dedicated palliative OPAT multidisciplinary team (MDT) in 2023 to address this gap. The service targets patients with life-limiting illness unlikely to survive more than 1 year without IV antibiotics, where no oral alternatives exist or previous oral regimens have failed. Specialist palliative care input is embedded into weekly OPAT MDTs, with a dedicated palliative MDT held monthly.

**Objectives:**

We conducted a retrospective review of the Royal Free Hospital’s palliative OPAT service between March 2023 and December 2024, focusing on patient demographics, diagnoses, time on therapy and outcomes.

**Methods:**

Patients accepted by the palliative OPAT MDT were identified from the OPAT database. Only those receiving IV antimicrobials were included. Demographics and clinical data were extracted from electronic patient records. Families of ten patients were invited to provide feedback via questionnaire; analysis is ongoing.

**Results:**

Nineteen patients were referred, with 17 included (records for two were inaccessible). At review, 14/17 (82%) had died. The average age was 63 years (range 27–91), with male to female ratio 11:6. Indications were grouped into: *Group 1 (n=10)*: incurable infections not amenable to surgery or oral therapy, including infected aortic grafts (*n*=4), infected biomass/abscess post-liver transplant (*n*=2), complex bone and joint infections (*n*=2), recurrent sepsis in primary immunodeficiency (*n*=1) and hydropneumothorax on cyclical IV therapy (*n*=1). *Group 2 (n=7)*: advanced terminal malignancies with superimposed infections requiring IV antibiotics, including HPB, gastrointestinal, lung, and bladder cancers. Five patients were treated empirically without positive microbiology available, guided by imaging, biomarkers, and clinical review. Modes of administration varied: 6 patients self-administered, 6 were supported by carers, 3 by district nurses, and 2 by hospice teams post-discharge. The mean duration on OPAT was 467 days (median 361; range 3–1288), substantially longer than in standard OPAT cohorts. One patient developed a *Pseudomonas aeruginosa* line infection; otherwise, no IV access complications occurred. Preliminary feedback from families are pending but general OPAT feedback expressed gratitude to the service and stated dignity, autonomy, and enhanced quality of life.

**Conclusions:**

The Royal Free palliative OPAT service demonstrates feasibility, safety, and meaningful patient benefit. High rates of self- or carer-administration (70.6%) highlight a patient-centred model that supports independence. The extended treatment durations, compared with conventional OPAT, underline the unique needs of this population with many patients living beyond their initial predicted prognosis. By enabling patients to remain at home, palliative OPAT improves quality of life, reduces inpatient stays, and may be cost-effective. Diagnostic patterns reflect the tertiary case mix particular to tertiary specialties at the royal free such as vascular, solid organ transplant and oncology. These findings support integrating palliative care into OPAT pathways and provide a structured model for delivery.

